# Evaluating enhanced recovery after surgery: time to cover new ground and discover the missing patient voice

**DOI:** 10.1186/s13741-020-00157-1

**Published:** 2020-09-14

**Authors:** Michael Nunns, Joseph B. John, John S. McGrath, Liz Shaw, Simon Briscoe, Jo Thompson Coon, Anthony Hemsley, Christopher J. Lovegrove, David Thomas, Michael G. Mythen, Rob Anderson

**Affiliations:** 1 Exeter HS&DR Evidence Synthesis Centre, Institute of Health Research, University of Exeter Medical School, University of Exeter, South Cloisters, St Luke’s Campus, Heavitree Road, Exeter, Devon EX1 2 LU UK; 2 Royal Devon & Exeter NHS Foundation Trust, Exeter, UK; 3 University of Exeter Medical School, Exeter, UK; 4 Department of Healthcare for Older People, Royal Devon & Exeter NHS Foundation Trust, Exeter, UK; 5 School of Health Professions, Faculty of Health & Human Sciences, Plymouth University, Plymouth, UK; 6 University College London Hospitals, National Institute of Health Research Biomedical Research Centre, London, UK

**Keywords:** Enhanced recovery after surgery, ERAS, ERP, Prehabilitation, Rehabilitation, Elective surgery, Meta-analysis, Recovery, Complications, Elderly

## Abstract

Multicomponent peri-operative interventions offer to accelerate patient recovery and improve cost-effectiveness. The recent National Institute of Health Research-commissioned evidence synthesis review by Nunns et al. considers the effectiveness and cost-effectiveness of all types of multicomponent interventions for older adults undergoing elective inpatient surgery. Enhanced recovery programmes (ERPs) were the most commonly evaluated intervention. An association between ERPs and decreased length of stay was observed, whilst complication rates and time to recovery were static or sometimes reduced. Important areas which lack research in the context of ERPs are patient-reported outcome measures, patients with complex needs and assessment of factors pertaining to successful ERP implementation. The next generation of ERP studies should seek to develop our understanding in these key areas.

## Main text

There is growing acceptance of the need to provide a standardised approach to peri-operative care, often tailored to the nature of the surgical intervention. Unwarranted variation is thought to impact both on clinical outcomes and patient safety. The cost of health care is growing exponentially and, at the same time, healthcare budgets have failed to keep pace. Enhanced recovery programmes (ERPs) are now established as multicomponent interventions that afford the opportunity to improve the quality of clinical care whilst reducing overall costs.

These ‘packages’ of care aimed at accelerating recovery and reducing length of stay (LOS) have become increasingly embedded across healthcare systems worldwide. Our recent systematic review and meta-analysis, commissioned by the National Institute for Health Research (NIHR), identified the broad range of interventions of this nature that have been studied in the UK and abroad (Nunns et al. 2019). It outlines how these interventions have been shown to improve recovery without significantly increasing the risk of complications or re-admissions. But importantly, it has furthered our understanding of which interventions, aspects of the ‘adoption’ process and key outcomes warrant rigorous assessment in future studies on account of the current paucity of relevant data available in the literature.

## The NIHR-commissioned evidence synthesis review

Our NIHR-commissioned systematic review was the first to consider the effectiveness and cost-effectiveness of all types of multicomponent interventions for older adults (mean or median age over 60) undergoing elective inpatient surgery (Nunns et al. 2019). The 73 studies prioritised for synthesis represented the most relevant and highest level of evidence available, based on the following criteria: randomised controlled trials (RCTs) from any high-income country and UK-based RCTs, controlled trials and uncontrolled studies.

As anticipated, lower limb arthroplasty and major colorectal surgery were the most commonly studied areas, representing 34% and 25% of studies respectively. Cardiac, pelvic, upper abdominal, thoracic, vascular and “various site” surgery accounted for the remainder. Six categories of multicomponent intervention were identified, including prehabilitation, rehabilitation and specialist wards and staff mix (see Table 1 for definitions). ERPs accounted for 67% of studies. ERP interventions were those describing components at multiple stages of the patient journey, including prior to hospital, throughout the admission and post-discharge, rather than only pre- or post-operative stages. Interventions classed as ERPs were also distinguished from other interventions by their nomenclature, encompassing those described in the literature as enhanced recovery after surgery (ERAS), ‘fast-track’, ‘accelerated recovery’ and other synonymous terms.

**Table 1:**
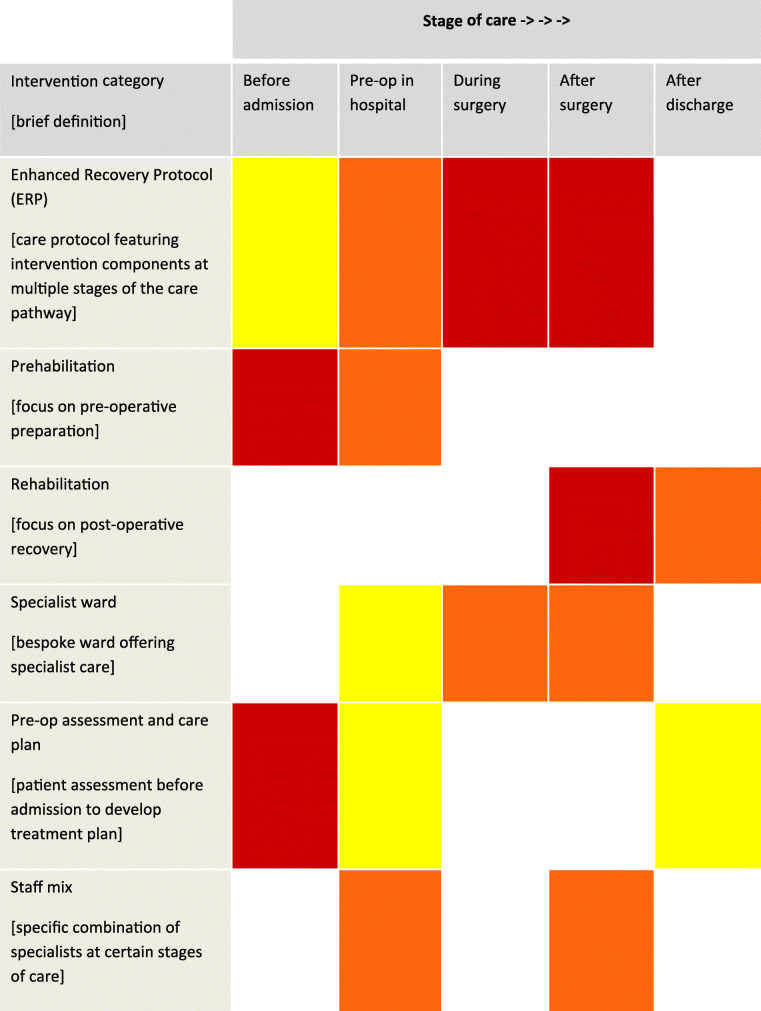
‘Heat map’ showing when interventions components were typically delivered for each intervention type

Yellow = low concentration of components, red = high concentration of components

We performed meta-analyses only where clusters of similar interventions, comparators and outcomes were available. Studies that were not eligible for meta-analysis were included in a narrative synthesis.

## The findings

Our broad systematic review confirmed the finding that multicomponent interventions, of any category, have an overall beneficial effect on one or more clinical or patient-reported outcomes and that they rarely lead to any inferior outcomes, in terms of what is published. As one might expect, the most consistent evidence from international RCTs associates ERPs with reduced LOS, particularly after colorectal surgery and upper abdominal surgery. Physical recovery, such as achievement of pain control, mobilisation goals and restoration of gastro-intestinal function, occurred earlier with ERPs in colorectal surgery. The available evidence from upper abdominal surgery suggested that patients exhibited reduced odds of sustaining complications with ERPs, albeit from a smaller body of literature assessing five patient groups. Evidence in other types of surgery was limited by small numbers of studies containing data that could not be synthesised and thus the focus of our discussion is predominantly on ERPs. Numerous international RCTs reported improved outcomes with ERP and prehabilitation, but were not amenable to meta-analysis either due to different comparators or outcomes. There were relatively few studies of intervention types other than ERP and prehabilitation, precluding meta-analysis and firm conclusions about their effectiveness.

UK evidence reflected the international findings. It was similarly dominated by ERP and, to a lesser extent, prehabilitation evaluations in colorectal surgery and lower limb arthroplasty. Meta-analysis of seven studies of ERP in lower limb arthroplasty indicated a 4-day reduction in LOS, and reduced LOS was observed in those patients undergoing upper abdominal surgery ERPs. A reduction or equivalence in LOS and complications occurred with colorectal ERPs. Various alternative markers of recovery improved across numerous UK studies.

## Key gaps

In terms of future improvements in clinical care, the key messages may lie in ‘what was not seen’ in the literature. The published evidence is awash with pre- and post-intervention case series often with a lack of contemporaneous control groups. The published findings are also limited by lack of rigour when defining interventions or outcomes. For example, LOS was inconsistently defined ranging from the time from admission to discharge, to the admission duration after surgery or simply the total time in hospital including readmissions. Over 40% of studies offered no clear LOS definition, limiting comparability between these. Only 12% of studies addressed either the reliability or validity of the primary outcome and comparator groups were often poorly reported. It was frequently difficult to determine which pathway components were only present in intervention groups and often the comparator was not described at all. This limits the extent to which studies can be replicated, compared and their methods adopted in practice.

Longer-term patient outcomes were almost entirely unmeasured and this represents one of the greatest unmet needs in ERP research. Only two studies investigated outcomes at 12 months. The majority ended their outcomes assessment 30 days after discharge and these were almost exclusively concerned with mortality, readmissions and complications. Patient-reported outcomes or experience measures (PROMs and PREMs) were not utilised and there is a need to study the patient-facing benefits of ERPs. We cannot say with any confidence that patient experience is improved by these interventions or that patients would like to see their LOS reduce further. There are pragmatic reasons to hypothesise that patient-experience may be improved, based on improved information-sharing, goal setting and shared decision-making, but our included studies offered no reliable evidence to substantiate this. Whilst there is evidence about patient satisfaction and experience (e.g. Hepner et al. 2004), future trials of ERPs and other multicomponent interventions in older adults require concurrent evaluation of patient experience. Our wide search strategy also confirmed that there is a lack of rigorous evaluation of the wider impact of reduced LOS on primary and social care systems. Only six studies assessed additional care after discharge, and the discharge destination was rarely reported.

Studies frequently excluded patients over a certain age or with the potential to experience complex needs. A number also chose to exclude ‘outliers’ or patients experiencing severe complications. Patients with risk factors for delirium were excluded from 18 studies despite delirium being a risk in many over 60-year-olds undergoing surgery. In contrast, a small number of studies (*n* = 7) selected individuals who had multi-morbidities or were at greatest risk of post-operative complications or of developing complex care needs.

Finally, cost-effectiveness evidence was derived from only 15 studies and these studies were highly heterogeneous in terms of population, intervention and location. Whilst there was a general suggestion that interventions lead to cost savings, findings were often the result of basic alignment with daily costs, and not the result of rigorously performed economic evaluations.

## Evaluating ERPs—future requirements

For colorectal surgery and lower limb arthroplasty, it can be strongly argued that further evaluations measuring the effectiveness of ERP interventions at reducing LOS, complications and re-admissions are not required. Future studies in these specialties would potentially add greater value if they had a new focus on implementation science, scaling up of adoption and the assessment of longer-term outcomes. In specialty areas where ERP effectiveness is less proven, further work should prioritise wider outcomes over longer time periods. All specialty areas have a pressing need to include outcomes that are more directly linked to the patient—including PROMs, PREM and the effects on physical activity and mental health. The effect of earlier post-operative discharge on the broader health and social care system requires elucidation, including comprehensive economic evaluation. Multicomponent interventions other than ERPs also require further evaluation, given the relative lack of quality evidence identified in these areas. Our report also offers recommendations for improving the academic quality of future studies, including adopting clear definitions of variables and methods for presenting data. Standardised variance statistics should also be included in future works to allow meta-analysis and maximise impact.

For ERP, there was no core configuration of intervention components that conveyed superiority for the study populations included in this review. Similar LOS improvements were realised with a common lack of detrimental outcomes. This suggests that endeavours to find the ‘ideal protocol’ are less important than the act of whole-team engagement in scrutiny and measurement of the patient pathway. A gap in understanding is the level of compliance with which interventions are implemented. Simpson et al. have observed a weak dose-response relationship between ERP protocol adherence and decreased LOS (Simpson et al. 2015). Implementation science could help us to characterise this relationship further by focusing on understanding the qualitative experiences of patients and staff. The multicomponent nature of ERP, and its involvement of numerous stakeholders, introduces potential for variation in the degree of uptake of pathways. As such, understanding implementation should be a further research priority.

With an ageing and increasingly co-morbid population, a final recommendation is that future studies evaluating multicomponent interventions should embrace the complexities of the older adult population. Studies were frequently observed to be selective against complex needs to the point that the UK elective surgical patient population is incompletely represented. Starks and colleagues actually observed their greatest improvement due to ERP in the oldest and most vulnerable patient cohorts who are most prone to long hospital admissions (Starks et al. 2014). Historically, there has been a perception that some patients may not be fit enough for ERP, but without good evidence, we cannot come to this conclusion and may be disadvantaging this group at large.

## Conclusion

Nunns et al. deliver a timely and detailed indication of our current understanding of multicomponent interventions for elderly patients undergoing elective surgery. The limits of our understanding are also defined; there is minimal knowledge of the effects of multicomponent interventions on PROMS, and most studies have excluded medically complex patients. In the future, measuring PROMs and including medically complex patients will require carefully designed studies. Understanding factors that allow multicomponent interventions to become truly embedded in practice will require a renewed approach in order to meaningfully connect the experiences of patients and staff, with measures of pathway adherence and outcomes. Ambitious expansion of our understanding of the effects of ERPs must be the future aim, rather than simply reinforcing what we already know.

## Data Availability

The datasets used and/or analysed during the current study are available from the corresponding author on reasonable request.

## Abbreviations

ERAS: Enhanced recovery after surgery
ERP: Enhanced recovery programme
LOS: Length of stay
NIHR: National Institute of Health Research
PREM: Patient-reported experience measure
PROM: Patient-reported outcome measure
RCT: Randomised controlled trial
